# Fecal transplant modifies urine chemistry risk factors for urinary stone disease

**DOI:** 10.14814/phy2.14012

**Published:** 2019-02-21

**Authors:** Joshua M. Stern, Marcia Urban‐Maldonado, Mykhaylo Usyk, Ignacio Granja, Daniel Schoenfeld, Kelvin P. Davies, Ilir Agalliu, John Asplin, Robert Burk, Sylvia O. Suadicani

**Affiliations:** ^1^ Department of Urology Albert Einstein College of Medicine Bronx New York; ^2^ Department of Microbiology Albert Einstein College of Medicine Bronx New York; ^3^ Litholink Corporation Chicago Illinois; ^4^ Department of Epidemiology Albert Einstein College of Medicine Bronx New York

**Keywords:** Calcium, gut microbiome, kidney stones, oxalate, urinary stone disease

## Abstract

Urinary stone disease (USD) is a major health concern. There is a need for new treatment modalities. Recently, our group provided evidence for an association between the GMB composition and USD. The accessibility of the Gut Microbiome (GMB) makes it an attractive target for investigation and therefore, in these studies we have evaluated the extent to which the whole gut microbial community in fecal transplants can affect urinary stone risk parameters in an animal model. Fresh fecal pellets were collected from Zucker lean rats, homogenized in PBS (100 mg/mL), filtered through a 70 *μ*m strainer and then orally gavaged into C57BL/6NTac germ‐free mice. Twenty‐four hours urine collections and GMB analysis were performed over time for 1 month. Kidney and gut tissue were harvested from transplanted mice for western blot analysis of expression levels of the Slc26a6 transporter involved in oxalate balance. Urinary calcium decreased after fecal transplant by 55% (*P* < 0.001). Urinary oxalate levels were on average 24% lower than baseline levels (*P* < 0.001). Clostridiaceae family was negatively correlated with urinary oxalate at 4 weeks after transplant (*r* = −0.83, *P* < 0.01). There was a 0.6 unit average increase in urinary pH from a baseline of 5.85 (SE ± 0.028) to 6.49 (SE ± 0.04) (*P* < 0.001) after transplant. There was a concomitant 29% increase in gastrointestinal alkali absorption (*P* < 0.001) 4‐weeks after fecal transplant. Slc26a6 expression increased by 90% in the cecum after transplant. Our results suggest that the gut microbiome may impact metabolism, alters urinary chemistry, and thereby may influence USD; the accessibility of the GMB can potentially be leveraged for therapeutic interventions.

## Introduction

Urinary Stone Disease (USD) is a growing public health burden in the United States, associated with increased risks of kidney function loss (Alexander et al. 2012), heart disease (Ferraro et al. 2013), and bone fracture (Melton et al. 1998) that results in annual healthcare costs exceeding $10 billion (Litwin and Saigal 2012). Recent advances in human Gut Microbiome (GMB) sequencing have led to innovative breakthroughs that describe its relationship to important human health outcomes such as asthma, inflammatory bowel disease, and cardiovascular disease (Black et al. 2015; Knoll et al. 2016; Butto and Haller 2017). Recently, our group provided evidence for an association between the GMB composition and USD (Stern et al. 2016).

A seminal report demonstrating that murine obesity could be transferred by fecal transplant (Turnbaugh et al. 2006) established the important role that the GMB has on metabolic regulation (Larsen et al. 2010; Wang et al. 2011; Vrieze et al. 2012; Everard and Cani 2013). As nearly 80% of stones contain oxalate, and because of the ability of *Oxalobacter formigenes (OF)* to metabolize oxalate, multiple studies have evaluated the potential for this single species to lower urinary oxalate (Siener et al. 2013; Holmes et al. 2016). Nonetheless, experimental *OF* colonization appears temporary, may be dependent on a narrow concentration of intraluminal calcium, oxalate, and pH (Hatch et al. 2011), and was recently found not to lower urinary oxalate compared to placebo in a Phase III clinical trial (Milliner et al. 2017). The lack of success of selective transplant of a single *OF* species has led other groups to investigate the effect of whole community fecal transplant from animal models fed a high oxalate diet, which appears to be more effective in lowering urinary oxalate (Miller et al. 2017). Overall, there is increasing evidence that microbial dynamics relies on a balance of cooperation and competition within the whole microbial ecological environment which is likely to play a role in the relationship between USD and the microbiome (Rakoff‐Nahoum et al. 2016). Recent data have also associated antibiotic use with risk of USD (Tasian et al. 2018). Therefore, in these studies we have investigated the extent to which the whole microbial community in fecal transplants can affect urinary stone risk parameters. Our results suggest that the microbiome could impact the metabolic status of patients and thereby contribute to USD; the accessibility of the GMB can potentially be leveraged for therapeutic interventions (Guthrie et al. 2017).

## Methods

### Fecal transplant

Two healthy lean Zucker donor rat (male 300–350 g; Charles River Laboratories, Wilmington, MA) and nine recipient germ‐free mice (male 8‐week old C57BL/6NTac; Taconic Biosciences, Rensselaer, NY) were used in the GMB transfer studies. Rats were chosen as fecal donors because of the closer similarities of their GMB with that of humans (Manichanh et al. 2010). Upon arrival at our institution, the germ‐free mice were directly transferred to flexible film isolators in our Gnotobiotic Facility at Einstein's Animal Institute. One week after arrival and acclimation, the germ‐free status of the mice was confirmed by quantification of copy numbers of 16S ribosomal DNA in the fecal pellets. The germ‐free mice were then transferred to a step‐down room in our Gnotobiotic Facility and individually housed in order to quantify and control dietary intake. At the time of fecal transfer, fresh fecal pellets were collected from the Zucker donor rat, homogenized in phosphate buffered saline (100 mg/mL; 1X PBS) and filtered through a 70 *μ*m strainer. Immediately after preparation 200 *μ*L of the filtrate was given by oral gavage to recipient germ‐free mice, as previously reported (Turnbaugh et al. 2006; Zhang et al. 2015). GMB analysis and 24‐h urinary chemistry was performed with samples collected from the donor rats immediately after fecal donation, and with the recipient mice at baseline (prior to fecal transplant) and at 1, 2, 3, and 4‐weeks after fecal transplant. Urine and fecal pellets were collected from individual animals placed in metabolic chambers. Body weight, food intake, and water intake were also measured at these time points. All mice were fed autoclaved sterile mouse chow. Fecal transplanted (*n* = 9) and control group germ‐free mice (*n* = 9) were sex and age matched and maintained in insulators in our Gnotobiotic facility for the 4‐weeks duration of the experiment.

### Microbiome analyses

Methods for 16S rRNA gene amplification and sequencing were performed as previously described (Smith et al. 2012, 2016; Ghartey et al. 2014; Zhang et al. 2015; Stern et al. 2016; Usyk et al. 2017). Briefly, fecal samples were extracted for total DNA using the bead‐beating procedure of the MoBio PowerSoil DNA isolation kit (Qiagen laboratories, USA). The microbiome was characterized using unique barcoded primers for each sample that amplified the 16S rRNA V4 region. The PCR products were confirmed by gel analysis, pooled, isolated, and a library was created using the KAPA library preparation kit (Kapabiosystems, Wilmington MA) with TruSeq adapters. The DNA library containing barcoded DNA amplicons was sequenced on an Illumina MiSeq (Illumina Inc., San Diego, CA) at the Epigenomics and Genomics Core Facility at Einstein using a 300 bp paired‐end chemistry.

#### Bioinformatics

To process the Illumina reads, novobarcode (Hercus 2009) was used to demultiplex reads based on sample specific dual barcodes, and prinseq (Schmieder and Edwards 2011) was used to quality trim bases that had a PHRED score of 25 or less. VSEARCH (Rognes et al. 2016) was used to perform chimera detection and quality filtering. OTUs were created using closed reference selection with VSEARCH call by QIIME1.9 using a custom database, which contains reference sequences from Green‐Genes 13.8 (DeSantis et al. 2006), HOMD 14 (Chen et al. 2010), and the UNITE database (Abarenkov et al. 2010). Taxonomy was assigned using the uclust (Edgar 2010) (16S) and BLAST (Altschul et al. 1990) algorithms. Representative sequences were aligned using PyNAST (Caporaso et al. 2009) and phylogenic analyses were performed using FastTree 2.0 (Price et al. 2010). General community clustering was performed on the most abundant genera and species using ward.D2 hierarchical clustering based on sample‐to‐sample Euclidian distances. Visualization was accomplished with R 3.2.1 (Team 2013) and the *ggplot2* package (Wickham 2016). *β*‐diversity was assessed using weighted and unweighted unifrac distances. Significance was calculated using PERMANOVA employing the adonis function from the *vegan* package (Oksanen et al. 2007). *α*‐diversity was analyzed based on the Shannon and Chao1 metrics with significance determined by the Kruskal–Wallis test. Functional gene content was assessed using PICRUSt (Langille et al. 2013), a bioinformatics software package designed to predict metagenome functional content from marker genes (e.g., 16S rRNA).

### Urine collection

Two rounds of 24‐h urine collection were performed per donor rat and per recipient mice at each of the time points in the study. Voided urine was continuously collected in the metabolic chamber reservoir containing mineral oil (0.5 mL to minimize evaporation) and Thymol (1st round; unacidified samples) or mineral oil and 4N HCl (2nd round; 5/100 *μ*L urine; acidified sample). Urinary parameters analyzed included calcium, oxalate, uric acid, citrate, NH_4_, sulfate, pH, creatinine, phosphate, sodium, potassium, magnesium, urea nitrogen, supersaturation (SS) CaOx, SSUA, SSCaP. Urinary chemistry was performed by Litholink Corporation. Titratable acid excretion was calculated from urine pH, phosphorus, and creatinine as described by Lennon et al. (1966). GI alkali absorption is estimated: (Na + K + Ca + Mg)‐ (Cl + 1.8P) (Oh 1989).

### Western blotting

Fecal transplanted and age matched germ‐free control mice were euthanized in a CO_2_ chamber (to effect) at the end of the experiment (4‐week time point). The kidneys and the small and large intestines were harvested, placed in cold 1XPBS and connective tissues removed. Segments from the ileum (~3 cm from ileocecal junction), cecum (middle part), and colon were cut and the lumens thoroughly rinsed with 1XPBS. The tissues were then minced with a pair of fine scissors, flash frozen in N_2_, and stored at −80°C until processing. Protein expression levels of the Slc26a6 (A6) transporter, responsible for regulating oxalate secretion were quantified by western blotting, as we have previously described (Seref‐Ferlengez et al. 2016). Briefly, samples with equal protein concentration were resolved by SDS‐PAGE and transferred to nitrocellulose membranes (Whatman, Dassel, Germany). After 30 min incubation with blocking buffer containing 0.5% Tween‐20 (TBS‐T) and 2% nonfat dry milk, at room temperature, the membranes were incubated overnight at 4°C with the goat‐anti SLC26A6 middle region antibody (1:1,000; cat # OAEB02669, Aviva Systems Biology, CA) or with mouse‐anti GAPDH antibody (1:20,000 Fitzgerald, Acton, MA). Membranes were then incubated for 1 h at room temperature with respective horseradish peroxidade (HRP)‐conjugated donkey anti‐goat IgG (1:10,000; Santa Cruz, TX) and anti‐mouse IgG (1:10,000; Santa Cruz Biotechnology, TX). Protein bands were detected on the Azure c600 Bioanalytical Imaging System (Azure Biosystem, Dublin, CA) using the Immobilon Western detection kit (Millipore, MA). Densitometric analyses were performed using ImageJ (NIH) software. Measured intensities were first normalized by the GAPDH loading control and then by their respective average controls.

### Statistical analysis

We used the paired *t*‐test to compare averages before and after intervention with the fecal transplant. To take into account the repeated measurements of urinary parameters over a 4‐week period, we used a repeated measure analysis of variance (ANOVA) model to test differences in urinary parameters between baseline and each weekly treatment (1 through 4‐weeks). This method accounts for urinary measurements that are correlated within the same mouse over x time‐point. Bacterial species or genera that had an average prevalence of 1% or higher were correlated to changes in urinary composition between baseline and weeks 4 using the Spearman correlation coefficient. We also used Students *t*‐test to compare the average expression levels of receptors before and after intervention. All comparisons were two‐sided using a significance level of <0.05.

## Results

**Figure 1:**
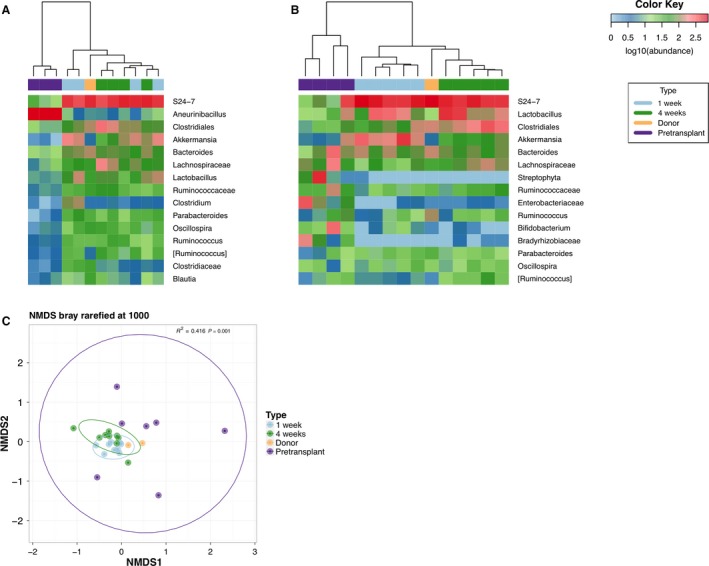
Fecal transplant into germ‐free mice. Two independent transplant experiments were performed with fecal pellets obtained from a single but different rat donor. Heat maps of the most abundant bacteria detected in the stool samples from (A) Rat donor 1 (yellow box) and from 4 fecal transplant recipient mice (note that at baseline in group A, *n* = 1 of the germ‐free pre‐transplanted mice did not have sufficient bacterial load to report and in group B *n* = 2 of the germ‐free pre‐transplanted mice did not have sufficient bacterial load to report), and from (B) Rat donor 2 (yellow box) and 5 recipient mice. Germ‐free recipient mice were sampled at baseline, 1 week and 4 weeks after fecal transplant. Hierarchical clustering in panels A and B indicate that both sets of mice acquired and maintained the donor's gut microbiome. (C) Community ordination using bray‐distances, visualized using non‐metric multidimensional scaling (NMDS) showed that there were significant community differences in the microbiome that could be explained using the transplant variables (*R* ^2^ = 0.416, *P* = 0.001). Prior to transplant the mice had a highly variable microbiome as indicated by the wide dispersion of samples across the NMDS plot, but formed tighter clusters surrounding the donors following transplant. Distribution of organisms at the genus level in stool samples are shown.

The germ‐free status of the mice before fecal transplant was confirmed by the presence of low fecal microbial reads, typical of a germ‐free GMB (Zhang et al. 2015) and that markedly increased after fecal transplant (FT) by oral gavage (Fig. 1A). Bacterial persistence at 1 and 4 weeks after FT is shown in Figure 1B and demonstrates efficient colonization by the donor GMB. The GMB of the two donor rats used in this study also clustered tightly (Fig. 1C).

**Figure 2:**
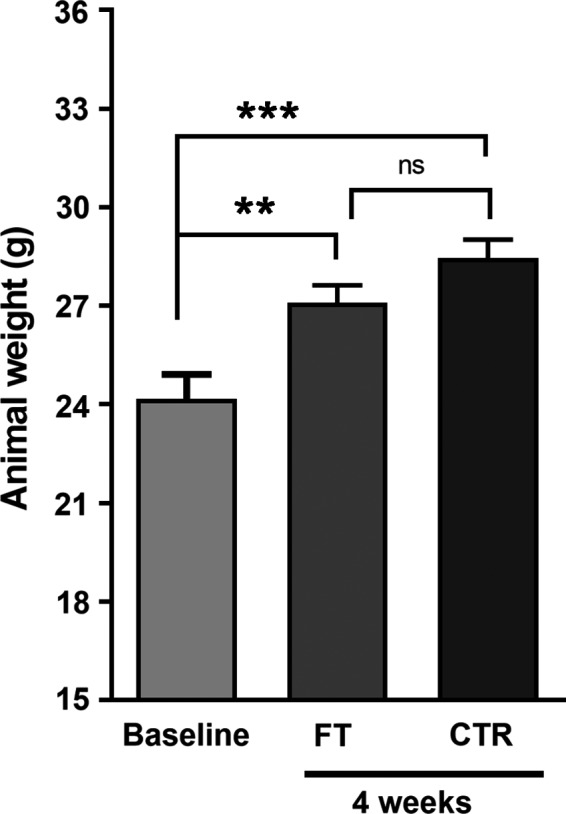
Body weight of germ‐free mice at baseline and at 4 weeks after receiving fecal transplant. Animals in both control (CTR) and fecal transplanted (FT) groups gained significant weight along the 4 weeks of the experiment when compared to baseline values. Body weight gain was not different between CTR and FT germ‐free mice. Data correspond to mean ± SEM; *n* = 9 per group; Student's *t*‐test: **P < 0.01 and ***P < 0.001.

**Figure 3:**
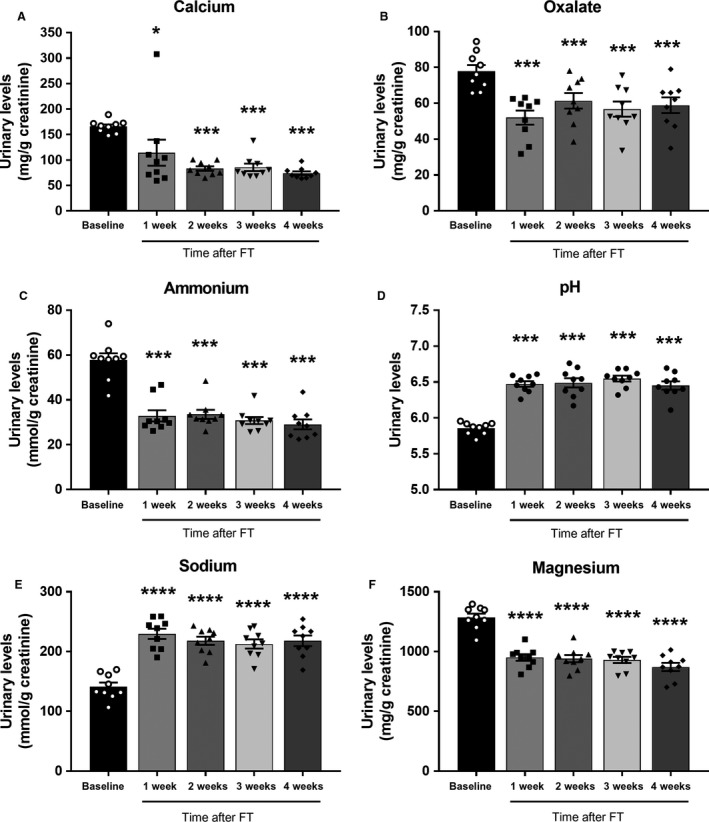
Urinary Chemistry at baseline, and at 1, 2, 3, and 4 weeks after fecal transplant. 24‐h urine parameters were normalized by creatinine (Cr). Data correspond to mean±SEM, *n* = 9 baseline and *n* = 9 subsequently transplanted mice. Statistical difference compared to baseline with pairwise comparison *P < 0.05; **P < 0.01; ***P < 0.001; ****P < 0.0001.

There was no difference in weight gain between the 4 week FT (*n* = 9) group and the 4 week age matched germ‐free control group (*n* = 9), respectively (Fig. 2). In addition, 24‐h urinary creatinine (Cr) remained at similar levels over the course of the experiment, with no statistical differences between baseline and at 4 weeks post FT (1.42 ± 0.06 mg/day and 1.39 ± 0.12 mg/day, respectively; *P* = 0.31). All 24‐h urinary parameters were then accordingly corrected for 24‐h creatinine (Fig. 3).

### Calcium oxalate metabolism

FT induced a significant reduction in urinary calcium (Ca) and oxalate (Ox) levels when compared to baseline levels (Fig. 3A and B). These changes were observed as early as 1‐week after FT and remained stable for the 4‐week duration of the study. As shown in Figure 3A, urinary Ca decreased after FT to reach values that were on average 55% lower at 4‐weeks post FT than those measured at baseline (*P* < 0.001). Repeated measure analysis showed no differences in urinary Ca levels between 2‐ and 3‐weeks (*P* = 0.82), 2‐ and 4‐weeks (*P* = 0.99), and 3‐ and 4‐weeks (*P* = 0.55) post‐transplant. At 4‐weeks after FT, urinary Ox levels were on average 24% lower than baseline levels (*P* < 0.001). Similar to the observations of Ca levels, this effect was seen rapidly at 1‐week post‐transplant and then stabilized for the remaining duration of the experiments (Fig. 3B). Repeated measure analysis also showed no differences in urinary Ox between 2‐ and 3‐weeks (*P* = 0.62), 2‐ and 4‐weeks (*P* = 0.94) and 3‐ and 4‐weeks (*P* = 0.96) after FT. Supersaturation (SS) of CaOx fell by 68% at 4‐weeks post‐transplant (*P* < 0.0001). Urinary Ca and Ox measured from 4‐week age‐matched germ‐free control animals largely mirrored the baseline levels of the germ‐free mice prior to transplant, thus indicating that changes in urinary chemistry were not secondary to aging (data not shown). However, in the case of Ox levels the average urinary excretion in the 4‐week age‐matched germ‐free control mice was 14% lower than the baseline values prior to transplant (*P* = 0.03).

These observed changes in urinary Ox were accompanied by changes in many of the prominently abundant bacteria that are not typically considered to be involved with Ox metabolism. At 4 weeks post‐FT, *Sutterella* genera was negatively associated with urinary Ox (*r* = −0.85, *P* < 0.01). Clostridiaceae family contain taxa known to be active oxalate degraders (Miller et al. 2014) and was also negatively correlated with urinary Ox at 4 weeks (*r* = −0.83, *P* < 0.01). Both Clostridiaceae and *Sutterella* genera were closely correlated with each other (*r* = 0.90, *P* < 0.01).

**Figure 4:**
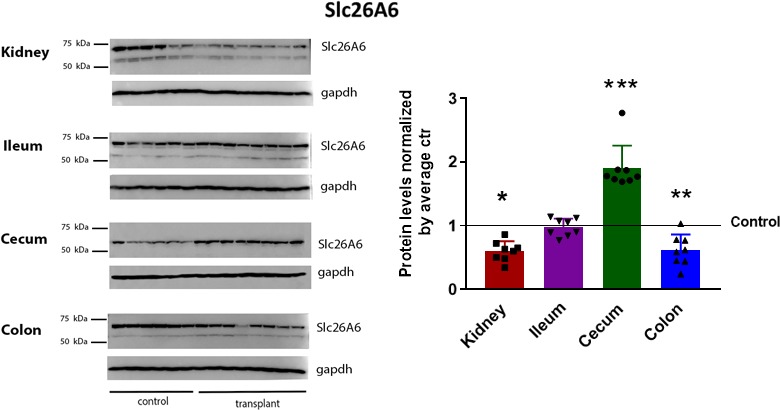
Fecal transplantation alters protein expression of Slc26A6 transporter involved in secretion of oxalate. (A) Western blot for the Slc26A6 transporter in kidney, ileum, cecum and colon tissues harvested from germ‐free age match controls and fecal transplanted mice at 4‐weeks after transplanted. Average Slc26A6 receptor protein levels, indicating that in fecal transplanted animals, Slc26A6 expression in the kidney decreased by 40.25% (*P* = 0.0137). There was no significant difference in expression observed in the ileum. Cecum Slc26A6 increased by 90% as compared to control (*P* = 0.0005) and colon Slc26A6 decreased by 38.50% (*P* = 0.0051) when compared to controls. Protein band intensities were first normalized by the GAPDH loading control and then by their respective controls and expressed as mean ± SEM; *n* = 6 age match controls and 8 fecal‐transplanted mice (1 mouse died prior to tissues collection); Student's *t*‐test compared to controls: *P < 0.05, **P < 0.01 and ***P < 0.001.

Based on our findings that GMB transfer is associated with a decrease in urinary CaOx, we performed western blot analyses of kidney, ileum, cecum, and colon tissue lysates from germ‐free age matched controls (*n* = 6) and 4‐weeks post‐transplant mice (*n* = 9) to investigate whether changes in urinary chemistry were related to altered expression of the Slc26a6 (A6) transporter, involved in the regulation of oxalate secretion. As shown in Figure 4, A6 protein expression in the kidney, cecum and colon were significantly and differentially influenced by the GMB. In association with the 24% decrease in urinary Ox observed at 4 weeks post‐FT, the levels of A6 in the kidney (where it is involved in the proximal tubular secretion of Ox) was 40.25% lower than those in controls (*P* = 0.013). There was no significant difference in expression observed in the ileum, whereas cecal A6 increased by 1.9‐fold (*P* = 0.0005) and colonic A6 decreased by 38.50% (*P* = 0.005) (Fig. 4A). Because we are aware that *OF* can influence A6 expression (Hatch et al. 2006), we ran a focused species level analysis and did not identify *OF,* however we do understand that with the addition of metagenomics *OF* is more often identified, yet still in very small abundance.

### Acid–base metabolism

Urine pH measured at baseline, and at 1‐, 2‐, 3‐, and 4‐weeks post‐transplant indicated that fecal transplant results in a significant reduction in renal acid excretion, suggesting a decrease in net acid load. There was a 0.6 unit average increase in urinary pH from a baseline of 5.85 (SE ± 0.028) to 6.49 (SE ± 0.04) (*P* < 0.001) at 4‐weeks after FT (Fig. 3D). The increase in pH was seen as early as 1‐week post‐FT and remained stable with no further changes during the 4‐week duration of the study. There was a 50% decrease in urinary ammonium (*P* < 0.0001) at 4 weeks post‐transplant and like the changes in pH, this change was identified as early as 1‐week and remained stable from weeks 1–4 after FT (Fig. 3C). There was a concomitant 29% increase in gastrointestinal alkali absorption (*P* < 0.001) 4‐weeks after FT. We were unable to calculate true renal net acid excretion (NAE) because we did not measure urine bicarbonate. NAE, however, does appear to fall after FT. This is reflected by a decrease in both components of the acid excretion, ammonium, and titratable acid. Urinary uric acid decreased by 22% from baseline at 4 weeks after FT (*P* = 0.06). Repeated measure analysis shows no inter‐week variation after the initial decrease observed at 1 week after FT. Urinary citrate increased by 31% from baseline at 1 week after FT (*P* < 0.001) and then progressively fell along at 2, 3‐weeks post‐transplant and at 4‐weeks there was no significant difference in citrate compared to baseline (*P* = 0.27). At 4‐weeks the age‐matched germ‐free controls had unchanged urinary chemistries from the baseline germ‐free mice, suggesting the changes noted in the FT mice were not due to aging or growth.

Correlation analysis demonstrated that *Clostridum* genus was inversely correlated with urinary ammonium (*r* = −0.82, *P* < 0.01) and with urinary uric acid (*r* = −0.83, *P* < 0.01).

### Nutritional changes

Urinary sodium increased by 34% from baseline to 4‐weeks post‐transplant (Fig. 3E, *P* < 0.0001). These changes were seen at 1‐week after transplant and remained stable over the remaining 4‐weeks. There were no differences seen in urinary sodium between weeks 2 and 3 (*P* = 0.97), weeks 2 and 4 (*P* = 1.0), or 3 and 4‐weeks (*P* = 0.93) after FT. Urinary magnesium decreased by 32.3% from baseline to 4 weeks after FT (Fig. 3F, *P* < 0.0001) and this change was seen as early as 1 week after FT, with no additional changes see from week 1 to 4 after FT.

## Discussion

For the first time we demonstrate that FT, transfer of a GMB from one animal to another, can impact the urinary parameters of the recipient animals. Furthermore, we demonstrate that these changes in urinary parameters correlate with changes in systemic expression of proteins involved in oxalate metabolism.

Early reports suggest a role for the GMB in USD (Hesse et al. 1999; Hatch et al. 2006; Barnett et al. 2016; Stern et al. 2016). Because of the ability of *Oxalobacter Formigenes (OF)* to metabolize oxalate, past studies evaluated the potential for this single species to lower urinary oxalate (Kaufman et al. 2008; Siener et al. 2013; Holmes et al. 2016). However, single species *OF* colonization appears to be temporary and may be dependent on a narrow concentration of intraluminal calcium, oxalate, and pH (Hatch et al. 2011). In addition, *OF* was recently found to be ineffective at lowering urinary oxalate compared to placebo in a Phase III clinical trial (Milliner et al. 2017). In contrast, Miller et al. (2017) recently demonstrated that whole community fecal transplant produced a more durable boost to oxalate metabolism then single species *OF* transplant. Given this data and our pilot study in USD patients (Stern et al. 2016) that suggests there is a unique GMB signature in these patients, we investigated in this study the impact that a whole community fecal transplant would have in altering the urinary chemistry and thus USD risk.

We observed that germ‐free mice experienced a significant and dramatic decline in urinary calcium with the introduction of a GMB. This finding contrasts with reports that in preterm infants and term infants urinary calcium is higher than that of older children and steadily decreases and becomes less variable with age (Sargent et al. 1993; Rockwell et al. 2008). This becomes clinically important in the subset of pre‐term infants that may develop nephrocalinosis secondary to high urinary calcium. The infant microbiome starts taking shape after delivery and organizes and establishes itself around 3‐months post‐partum. It is plausible, among other explanations as well, that the pre‐term child has an immature microbiome, similar to that of the germ‐free mouse, and that as one's GMB organizes urinary calcium then begins to normalize as is seen in infants as they age. The 55% decrease in urinary calcium seen at 1‐week after fecal transplant remained stable for 4 weeks and similarly, the GMB at 1 and 4‐weeks show stable clustering around the donor (Fig. 1C).

The changes in urinary chemistry observed in germ‐free mice after fecal transplant (Fig. 3) are widely recognized as important determinants of urinary stone risk (Sakhaee et al. 2012; Cheungpasitporn et al. 2016). The observed significant decrease in urinary calcium and oxalate seen at 4 weeks post‐transplant suggests that the GMB can influence CaOx balance. Given the current view that *OF* colonization may be integral to GMB metabolism of oxalate, we were intrigued that fecal transplant from a standard healthy animal with no identified *OF* on 16S sequencing, significantly lowered urinary oxalate imposing a 3.6‐fold decrease in CaOx super saturation. Several standard community organisms, however, have been correlated with urinary oxalate. We, like others (Suryavanshi et al. 2016) found *Sutterella* genus to be negatively correlated with oxalate. Clostridiaceae family contains many genera known to be active oxalate degraders (Miller et al. 2014) and was also negatively correlated with urinary oxalate at 4 weeks.

Both dietary and endogenously produced oxalate is excreted almost entirely in the urine. In the healthy state, oxalate homeostasis is thus a balance between intestinal secretion and absorption, and renal excretion. One transporter in particular, the Slc26A6, has been identified with an affinity for modulating oxalate flux. Slc26A6 is expressed in the intestine and kidney, and in the distal colon it promotes oxalate secretion (Yao et al. 2005; Jiang et al. 2006; Knauf et al. 2011; Freel et al. 2013). When Slc26a6 is knocked out, intestinal oxalate secretion decreases and urinary oxalate dramatically rises (Jiang et al. 2006). Interestingly *OF*, in addition to its intraluminal oxalate‐degrading capacity, has been shown to have a more complex interplay with bowel physiology by inducing intestinal oxalate secretion through interaction with Slc26a6 (Hatch et al. 2006; Arvans et al. 2017). Given the difficulties in sustaining *OF* colonization, it is clinically relevant that a whole community fecal transplant, as shown in this study from a GMB with undetectable levels of *OF,* was able to modulate protein expression of Slc26a6 in both the kidney and the colon with a concomitant 24% decrease in urinary oxalate. Such data, support the role of the whole GMB community and suggests that regulation of transporters and channels involved in calcium‐oxalate flux is modulated by the GMB and may not be driven by just one species. Modulation of intestinal transporter/channels levels responsible for CaOx balance with targeted GMB manipulation may represent and lead to a new line of therapeutic target to reduce urinary stone risk.

Given that intestinal alkali absorption is an established and important physiological process (Tang et al. 2015), it seems likely that the microbiome may influence its balance. Calculated GI alkali absorption significantly increased at 4 weeks after FT. The actual role the GMB in increasing alkali in this model is unclear, but certainly provocative. Nonetheless, the GMB, via short‐chain fatty acid production or other secretory functions, is key to maintenance of the gut mucosal barrier (Corfield 2018). Intestinal microbiota are also known to participate in purine metabolism. Of notable interest is that Clostridia have been shown to be capable of uric acid metabolism, and are able to use purines as sole carbon, nitrogen, and energy sources (Durre and Andreesen 1982; Hartwich et al. 2012). Correlation analysis demonstrated that *Clostridium* genus was inversely correlated with urinary ammonium and urinary uric acid at 4 weeks after FT.

This study has shortcomings. Not all animal models are translatable to the patient. The germ‐free animal does not make kidney stones and as such we used 24 h urine parameters as a surrogate endpoint for USD risk. Slc26a6 may not work alone, and in the future studies of other transporters and channels that modulate oxalate flux will be considered. While recognizing the potential for cross‐reaction of antibodies to yield false‐positive results, the specificity of various antibodies used to probe for Slc26a6 has been demonstrated in western blot data in the intestine and kidney from Slc26a6 knock‐out animals (Freel et al. 2006; Jiang et al. 2006). We are thus confident in the western blot analysis we present. Lastly, we recognize that understanding how serum pH and calcium change with the observed changes in urinary electrolytes following FT would be a valuable addition to this study. Furthermore, serum would allow a true calculation of fractional excretions of notable urinary parameters. Lack of such serum parameters somewhat limits our ability to fully understand the effects of the GMB on CaOx balance. In future studies, we hope to secure serum samples that will add to the findings presented here and thereby provide a broader view of the influence of the GMB on the relationship between urine, blood, and whole body electrolyte balance.

The increasing prevalence of kidney stones (Litwin and Saigal 2012) and the high stone recurrence rates (Scales et al. 2012; Tasian et al. 2016), despite dietary counseling and pharmacologic intervention, has motivated research to identify new treatment paradigms for kidney stone patients. Diet is considered a major contributing factor to USD (Pak 1998; Borghi et al. 2002; Taylor et al. 2004; Tracy et al. 2014) but the mechanisms underlying the association between diet and USD are poorly understood. It is likely that the GMB influences diet driven metabolism and as such has been linked to several medical conditions including kidney stones (Hesse et al. 1999; Hatch et al. 2006; Barnett et al. 2016; Stern et al. 2016). There is evidence that USD, similar to DM and obesity, may be associated with a unique GMB profile (Turnbaugh et al. 2006; Larsen et al. 2010; Stern et al. 2016). Furthermore, knowing that both DM and obesity can be influenced with fecal transplant combined with indications from our data that GMB transfer alters urinary chemistry provide the further rationale to continue to learn how to manipulate the microbiome to lower USD risk.

## Conflict of Interest

None declared.
